# Costs and Tradeoffs of Resistance and Tolerance to Belowground Herbivory in Potato

**DOI:** 10.1371/journal.pone.0169083

**Published:** 2017-01-17

**Authors:** Etzel Garrido, Maria Fernanda Díaz, Hugo Bernal, Carlos Eduardo Ñustez, Jennifer Thaler, Georg Jander, Katja Poveda

**Affiliations:** 1 Department of Entomology, Cornell University, Ithaca, New York, United States of America; 2 Fundación Biodiversa Colombia, Bogotá, Colombia; 3 Universidad Nacional de Colombia, Facultad de Agronomía, Bogotá, Colombia; 4 Boyce Thompson Institute for Plant Research, Ithaca, NY, United States of America; Helmholtz Zentrum Munchen Deutsches Forschungszentrum fur Umwelt und Gesundheit, GERMANY

## Abstract

The success of sustainable crop production depends on our ability to select or create varieties that can allocate resources to both growth and defence. However, breeding efforts have emphasized increases in yields but have partially neglected defence traits against pests. Estimating the costs of multiple defences against tuber herbivores and the tradeoffs among them, as well as understanding the relationship between yield and multiple defences is still unknown but relevant to both basic and applied ecology. Using twenty commercial potato varieties available in Colombia and the tuber herbivore *Tecia solanivora*, we tested whether high yielding varieties show a reduction in three types of defence: constitutive and induced resistance, as well as tolerance. Specifically, we determined (1) the costs in terms of yield of all three defences, (2) the possible tradeoffs among them, and (3) if oviposition preference was related to the expression of these defences. We detected no costs in terms of yield of constitutive and induced resistance to tuber damage. We did, however, find evidence of costs of being able to tolerate tuber herbivory. While we found no tradeoffs among any of the estimated defences, there was a positive correlation between aboveground compensatory growth and tolerance in terms of tuber production, suggesting that after damage there are no shifts in the allocation of resources from aboveground to belowground biomass. Finally, we found that females laid more eggs on those varieties with the lowest level of constitutive resistance. In conclusion our findings suggest that in potatoes, breeding for higher yields has not caused any reduction in constitutive or induced resistance to tuber damage. This is not the case for tolerance where those varieties with higher yields are also less likely to tolerate tuber damage. Given the high incidence of tuber pests in Colombia, selecting for higher tolerance could allow for high productivity in the presence of herbivores. Finding mechanisms to decouple the tolerance response from yield should be a new priority in potato breeding in Colombia to guarantee a higher yield in both the presence and absence of herbivores.

## Introduction

Sustainable crop production depends on our ability to decrease pest pressure while simultaneously increasing yield. One way to accomplish these goals is to identify and harness those traits that allow plants to naturally resist and tolerate herbivory without any decrement in yield. While resistance traits allow plants to limit or reduce the amount of damage they receive, tolerance traits allow plants to buffer the negative effect of damage in terms of fitness [1]. Resistance mechanisms can be constitutive or induced, whereas tolerance mechanisms are always triggered by herbivore attack. These defence mechanisms often co-occur within the same genotype or individual, and they are assumed to be costly, given that investment in defence should reduce the amount of resources available for growth and reproduction [2]. Therefore, negative associations among different defences have been predicted [3]. While a meta-analysis showed the absence of fitness costs for resistance [4], there is still no consensus about the fitness costs for tolerance with some studies detecting it [5–7] but others showing no cost [8–10]. As for the presence of tradeoffs among defences, meta-analyses have shown negative correlations between constitutive and induced resistance [11] but no tradeoffs between resistance and tolerance [12]. Most of these studies however have focused on the expression of defences to aboveground herbivores, and relatively little is still known about the costs and tradeoffs of plant defences to root or tuber herbivores [13].

Belowground herbivores play a key role in natural and agricultural ecosystems given their potential to shape plant communities [14], as well as aboveground arthropod communities [15]. In the past decade, the importance of studying belowground interactions and their consequences for the evolution of plant defences has been recognised, and the body of literature has been increasing steadily [13,16,17]. Nevertheless, most studies of belowground interactions have been focused on root herbivory and root chemical defences [18]. Little is still known about tuber-feeding herbivores and how these affect plant defences below- and aboveground. Also, the study of other types of defence, like tolerance to root herbivores [19,20] or tuber herbivores [21], has not received much attention.

Whether the damage is done above- or belowground, plant responses triggered by damage often occur both in the tissue originally attacked (induced local response) and in distant, yet unaffected, parts (induced systemic response). Among those traits that have been associated with local and systemic tolerance responses are changes in resource allocation patterns, architecture, photosynthetic activity and phenological patterns, as well as the activation of basal meristems and regrowth of lateral shoots and branches [22–24]. It is generally believed that induced defences, both local and systemic, are less costly than constitutive defences, thus saving energy under pathogen or insect-free conditions, although costs still arise when defences are activated following attack [25]. The ability of plants to systematically induce defences allows them to reduce or limit the amount of damage to additional organs. While the signaling pathways and potential benefits of induced systemic resistance have been widely investigated [26], we still lack much information about its costs and possible tradeoffs with other types of resistance (constitutive, induced local resistance) and tolerance traits.

Agricultural crops are appealing study systems for evaluating both the costs of different types of defence and the tradeoffs among them given that most crops have been subjected to strong positive selection for growth and yield [27]. Interestingly, for most crops studied, this increase in yield have come largely from the partitioning of photoassimilates to those organs of primary interest, and not from increases in photosynthetic rate [28,29]. Increases in yield therefore must be the result of a reallocation of plant resources, which include those resources available for defence. While some studies have found that domesticated plants have lower resistance levels compared to their wild relatives [30] others have not [31], suggesting that increases in yield do not always reduce resistance levels. Within a single crop, our hypothesis is that those varieties with higher yields will be less resistant, but not necessarily less tolerant, and thus herbivores could prefer to consume them. Although selecting for crop varieties that possess multiple defences may represent a more effective long-term pest management strategy than just selecting for higher resistance [32], we still lack a full understanding about the relationship between yield and the expression of multiple defence mechanisms.

By studying the response of twenty commercial potato varieties available in Colombia, including varieties of *Solanum tuberosum* and *S*. *phureja*, to the tuber damage of the Guatemalan potato moth *Tecia solanivora* we evaluated the following: (1) we examined the presence of genetic variation for both resistance (constitutive and induced) and tolerance to tuber herbivory; (2) we determined the costs associated with the expression of all types of defence and the possible tradeoffs among them, and (3) we assessed oviposition preference for all the varieties used and whether this preference was related to the expression of defence. Our data indicate no costs in terms of yield for the expression of constitutive or induced resistance. However, an increase in yield seems to come at an expense in tolerance. In the potato varieties studied, the expression of tolerance does not seem to result from a reallocation of resources from aboveground tissue to tuber mass, but it is probably the result of an increase in primary metabolism. The implications of our results for a more sustainable agricultural production are discussed.

## Materials and Methods

### Study system

Potato (*S*. *tuberosum* L.) is the world’s fourth most important food crop after maize, rice and wheat. Plants are vegetatively propagated from a single tuber or a piece of it. Depending on the variety, the new plant can grow around 60 cm and produce 5–20 new tubers. The Guatemalan potato moth, *T*. *solanivora*, Povolny 1973 (Lepidoptera: Gelechiidae) is considered one of the most damaging insect herbivores of crop potatoes in several regions of Latin America [33]. Females lay their eggs either in soil cracks, directly on the tubers, or at the base of the stem. Upon hatching, the larvae of this specialized herbivore bore into the tubers, with the potential to completely destroy a crop [33,34]. Twenty commercial potato varieties available in Colombia were included in the study: five varieties belonging to the species *S*. *phureja* (Criolla Colombia, Criolla Galeras, Criolla Guaneña, Criolla Latina and Criolla Paisa) and 15 varieties of *S*. *tuberosum* (Betina, Capiro, Esmeralda, Monserrate, Nevada, Parda Pastusa, Pastusa Suprema, Punto Azúl, Puracé, Roja Nariño, Rubí, Toquerreña, Única, V1 and Yungay).

### Plants, insects and growing conditions

All potato varieties were planted between March 2nd and April 13th of 2012 at the “San Jorge” experimental station of the Instituto Colombiano Agropecuario (ICA) located at 2800 m above sea level in the municipality of Soacha, Cundinamarca, Colombia. All potato plants and moths used for this experiments came from research institutions in Colombia and were not taken from the field. Tubers obtained from the breeding program (“Grupo de Investigacion en Papa”) of the Universidad Nacional de Colombia were planted in 2 gallon plastic bags filled with field soil from the region and fertilized with 15 g of N:P:K 10:20:20 (Ecofertil). No samples were taken from the field but obtained from the breeding program with permission from the owners. Thirty plants per variety were initially planted and this was repeated twice with an interval of three weeks for a total of ninety plants per variety. When most plants started to flower (which is a good indication of tuberization) they were transported from the experimental station to the greenhouses of the Agronomy Department of the Universidad Nacional de Colombia to perform the experiments. Adults and larvae of *T*. *solanivora* for all of the experiments came from a colony maintained in the Entomology laboratory of the Universidad Nacional de Colombia.

### Yield, tolerance and compensatory growth

Thirty-two plants per variety were used for these measurements. All of these plants were placed in the greenhouse in a randomized manner. One tuber per plant was carefully dug up, taking care not to sever or damage its stolon. These individual tubers, hereafter called focal tubers, were left at soil level and covered with a black cloth to prevent greening and other potential changes to primary and secondary metabolism [35]. Immediately after the focal tubers were chosen, half the plants of each variety received a damage treatment that consisted of placing ten neonate larvae on the surface of the focal tubers using a brush and again covering the tuber with the black cloth. Twenty-one days after the application of the damage treatment, all the plants were harvested. Aboveground biomass per plant was measured after drying the tissue in a drying oven at 60°C for three days. Tubers were separated into focal tubers and systemic tubers and then counted and weighed. At the time of harvest, all the tubers were checked for damage to verify that only focal tubers from the damage treatment were damaged. If a control plant presented damage on its focal tuber then it was considered as a replicate of the damage treatment. All plants that presented damage on any systemic tuber were not taken into account for statistical analyses. Yield was measured as the fresh weight of the undamaged tubers. Tolerance to the tuber-herbivore was then calculated as the relative difference in yield between the damaged and control plants. To test whether there is a tradeoff in the allocation to above- and belowground biomass (*i*.*e*. allocation to shoots vs tuber weight, in this study), we estimated the aboveground compensatory growth of the plants as the difference in aboveground dry-biomass between control and damaged plants.

### Resistance estimations

For plants belonging to the damage treatment, the local and one systemic tuber was used for bioassays. For plants in the control treatment, just one systemic tuber was used for bioassays. For all of the bioassays, tubers were first weighed and then individually placed in plastic cups (Solo® Clear Cup, 9 oz) containing fine sand and kept in the dark at 22°C. To each tuber, ten neonate larvae were added and left to develop inside the tuber until they started pupating in the sand. Emergence of pupae was recorded every two days until no more pupae came out. The number of emerged pupae allowed us to measure the mortality rate of the larvae in each single tuber as our measure for resistance. Constitutive resistance was estimated as the mortality of larvae placed in the tubers of the control treatment. Induced resistance was estimated as the difference between the mortality of damaged plants and control plants and was measured for focal tubers (local induction), as well as for systemic tubers (systemic induction). This metric of induced resistance has been considered the best when testing for costs of induced defences and for tradeoffs between constitutive and induced defences [36].

### Oviposition assays

To determine the oviposition preference of *T*. *solanivora* for the different potato varieties, we performed a non-choice oviposition experiment. Fifteen flowering plants per variety were transported to the greenhouses at the Universidad Nacional de Colombia to start the experiment. All the plants were placed randomly inside the greenhouse. After three days in the greenhouse, all plants were placed inside mesh bags (“Breather sleeves” by Palm Tree Packaging, Apopka, FL) and three pairs of virgin adults were placed inside each bag and allowed to reproduce and oviposit for one week. All of the eggs laid at the base of the stem, near or on the soil, and on the bags were then counted to have a proxy of preference.

### Data analyses

Three varieties (Parda Pastusa, Punto Azúl and Toquerreña) of *S*. *tuberosum* were eliminated from our analyses because the number of replicates per treatment at the end of the experiments was less than four. The presence of genetic (varietal) variation in the expression of all the types of defence measured was analyzed with a nested ANOVA using species and varieties nested within the species as categorical factors, tuber mass was included as a covariate for the resistance analyses because previous data have shown its effect on pupal mass [37]. Given the clear differences in resistance and tolerance between the two potato species (*see* Results, Table 2), we decided to standardize the values of defence by species to avoid a species bias in our analysis when determining the presence or absence of costs of defence in terms of yield and tradeoffs among defences. The standardization of the values was done with the formula $x_{i}–\bar{x}/\sigma$, where *x*_*i*_ represent each individual value and $\bar{x}$ and *σ* stand for the mean and standard deviation of each species. This standardization also reduces the covariation between the fitness in control and damaged plants, thus the estimation of the costs of tolerance is less biased [8]. To determine whether there are potential costs in terms of yield due to the expression of tolerance and resistance, Pearson correlations were calculated between the mean standardized values of each of our defence traits per variety and the mean standardized values of yield per variety in the absence of damage (control plants). The presence of tradeoffs among each of our defence traits per variety and the possible tradeoff between tolerance and aboveground compensatory growth were again assessed using Pearson correlations. Finally, we evaluated the effects of plant defence on the number of eggs laid per plant (*i*.*e*. a proxy of preference) with an ANOVA where the standardized mean values of resistance and tolerance per variety were included as factors. All statistical analyses were performed using R [38].

## Results

Overall, there were differences among the two species in constitutive resistance, locally induced resistance, and tolerance (Table 1). Larvae developing on *S*. *phureja* were 67% more likely to survive than larvae developing on *S*. *tuberosum*, indicating a higher constitutive resistance and/or a lower nutritive status of the latter species. On the other hand, the locally induced resistance was twice as high in *S*. *phureja* compared to *S*. *tuberosum* while the tolerance response of *S*. *phureja* was 15% higher (Table 2). We also detected genetic (varietal) variation for all of the types of defence measured, except for locally induced resistance (see Tables 1 and 2).

**Table 1:** Differences among species and genetic variation in the expression of different types of defence in commercial varieties of potato in response to the damage by the specialist tuber moth *T*. *solanivora*.

	*Sources of variation*								
	Species			Variety (Species)			Tuber Mass		
	*F*	*df*	*P*	*F*	*df*	*P*	*F*	*df*	*P*
**Resistance**									
*Constitutive*	120.25	1,196	**< 0.001**	1.97	15,196	**0.016**	0.76	1,196	0.383
*Locally Induced*	33.77	1,126	**< 0.001**	1.21	15,126	0.267	0.26	1,126	0.613
*Systemically Induced*	0.45	1,140	0.505	1.96	15,140	**0.429**	1.01	1,140	0.299
**Tolerance**	7.92	1,149	**0.005**	1.64	15,149	**0.043**	na	na	na

For all the analyses, variety was considered nested within species while tuber mass was included as a covariate only for the analyses of resistance. Values in bold are significant at a 5% level. na: not applicable.

**Table 2:** Mean values ± SE of different types of defence in commercial varieties of potato in response to the specialist tuber moth *T*. *solanivora*.

		Resistance		Tolerance
	*constitutive*	*locally induced*	*systematically induced*	
*Solanum phureja*	0.583 ± 0.028^B^	0.164 ± 0.034^A^	0.020 ± 0.029^A^	0.017 ± 0.032^A^
Criolla Colombia	0.550 ± 0.058^b^	0.277 ± 0.094^a^	0.097 ± 0.062^a^	-0.171 ± 0.036^b^
Criolla Galeras	0.630 ± 0.080^a^	0.163 ± 0.068^a^	0.070 ± 0.054^a^	-0.010 ± 0.057^b^
Criolla Guaneña	0.586 ± 0.069^a^	0.130 ± 0.067^a^	-0.101 ± 0.073^b^	0.147 ± 0.081^a^
Criolla Latina	0.567 ± 0.043^a^	0.173 ± 0.087^a^	-0.095 ± 0.064^b^	0.180 ± 0.075^a^
Criolla Paisa	0.586 ± 0.065^a^	0.101 ± 0.073^a^	0.114 ± 0.058^a^	-0.029 ± 0.080^b^
*Solanum tuberosum*	0.864 ± 0.012^A^	-0.068 ± 0.023^B^	-0.007 ± 0.017^A^	-0.107 ± 0.031^B^
Betina	0.807 ± 0.043^b^	0.043 ± 0.076^a^	0.110 ± 0.040^a^	-0.200 ± 0.145^b^
Capiro	0.958 ± 0.015^a^	-0.128 ± 0.059^a^	0.012 ± 0.021^a^	-0.063 ± 0.099^b^
Esmeralda	0.921 ± 0.021^a^	-0.064 ± 0.061^a^	-0.021 ± 0.044^a^	-0.193 ± 0.071^b^
Monserrate	0.867 ± 0.044^a^	-0.094 ± 0.080^a^	0.015 ± 0.033^a^	-0.038 ± 0.083^b^
Nevada	0.846 ± 0.049^a^	0.087 ± 0.033^a^	0.087 ± 0.033^a^	0.141 ± 0.041^a^
Pastusa Suprema	0.822 ± 0.043^a^	-0.022 ± 0.082^a^	-0.089 ± 0.105^a^	-0.097 ± 0.085^b^
Puracé	0.814 ± 0.043^a^	-0.081 ± 0.072^a^	0.069 ± 0.060^a^	-0.201 ± 0.144^b^
Roja Nariño	0.792 ± 0.050^b^	-0.042 ± 0.054^a^	0.045 ± 0.032^a^	-0.070 ± 0.070^b^
Rubí	0.808 ± 0.049^a^	0.029 ± 0.059^a^	-0.124 ± 0.059^b^	-0.196 ± 0.089^b^
Única	0.942 ± 0.023^a^	-0.042 ± 0.032^a^	-0.022 ± 0.080^a^	-0.164 ± 0.101^b^
V1	0.801 ± 0.045^a^	-0.330 ± 0.103^a^	0.101 ± 0.063^a^	-0.123 ± 0.188^b^
Yungay	0.971 ± 0.013^a^	-0.231 ± 0.163^a^	-0.051 ± 0.080^a^	0.039 ± 0.144^ab^

All types of resistance were measured in terms of larval mortality (%), tolerance was measured in terms of tuber weight (g). Different letters indicate differences following a Tukey–Kramer test: upper case letters denote differences between the two potato species, while lower case letters show differences within each species.

**Fig 1:**
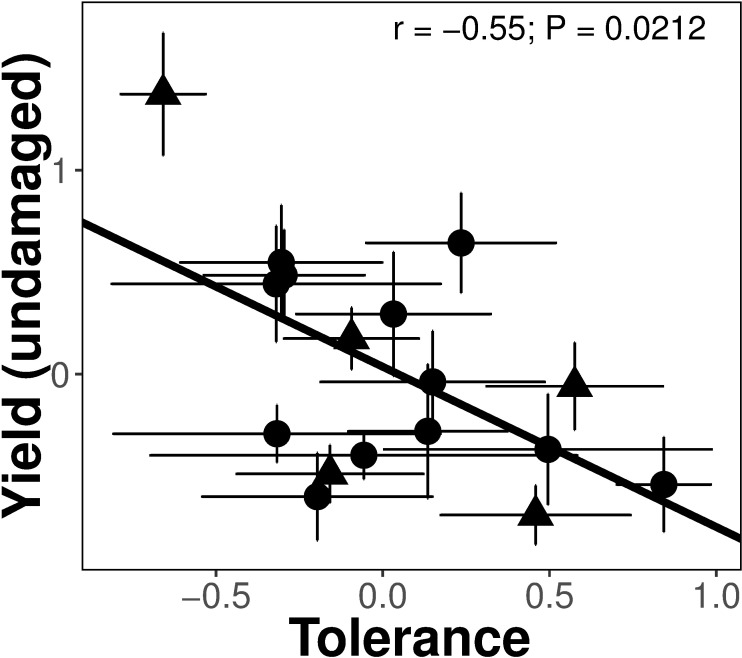
Cost in terms of yield of tolerance to the damage by the specialist tuber moth *T*. *solanivora*. Points represent mean values ± SE per variety. Shapes correspond to different potato species (circle: *S*. *tuberosum*, triangle: *S*. *phureja*).

**Fig 2:**
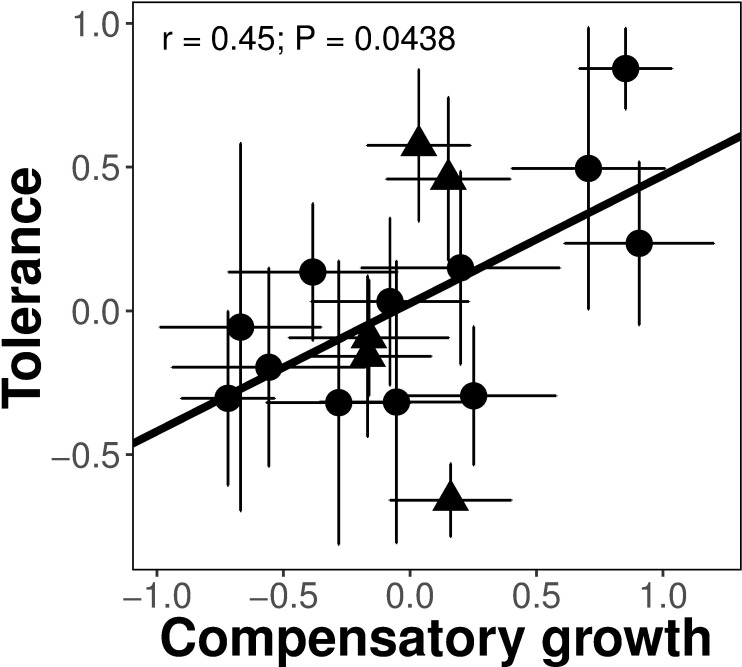
Correlation between compensatory growth and tolerance among different potato varieties in response to damage by the specialist tuber moth *T*. *solanivora*. A positive correlation suggests no tradeoff in the allocation for above- and belowground biomass after damage. Points represent mean values ± SE per variety. Shapes correspond to different potato species (circle: *S*. *tuberosum*, triangle: *S*. *phureja*).

After standardizing the data for the differences among the two species, we did not detect any costs in terms of yield of any type of resistance measured (Table 3). We found, however, evidence of a cost of tolerance (Table 3). That is, a higher expression of tolerance was negatively correlated with yield in the absence of herbivores (r = -0.55, *P* = 0.0212; Fig 1). We also did not detect tradeoffs between constitutive and induced resistance, as well as between tolerance and any type of resistance (see Table 4). Interestingly, there was a positive correlation between tolerance and compensatory growth (r = 0.45; *P* = 0.0438) suggesting no re-allocation of resources for below- and aboveground biomass after damage (Fig 2). Finally, while there were marginal differences in the number of eggs laid among all the potato varieties (*F*_18,203_ = 1.65; *P* = 0.0503), we found that females laid twice as many eggs on *S*. *phureja* varieties (*F*_1, 206_ = 19.15; *P* < 0.0001) (see Table 5), which express lower levels of constitutive resistance. There were however, no effects of any type of defence on the number of eggs laid per plant (all *F* < 1.53).

**Table 3:** Costs of different types of defence in potato in response to the damage by the specialist tuber moth *T*. *solanivora*.

*Type of defence*	*Yield* (in the absence of herbivory)
**Resistance**	
*Constitutive*	-0.17
*Locally Induced*	0.37
*Systematically Induced*	-0.06
**Tolerance**	**-0.55**

Costs are expressed as negative correlations between defence and yield in the absence of herbivory. Pearson correlation values are shown. Values in bold are significant at a 5% level.

**Table 4:** Tradeoffs among defences in potato in response to the damage by the specialist tuber moth *T*. *solanivora*.

		Resistance			Tolerance
		*Constitutive*	*Locally Induced*	*Systemically Induced*	
**Resistance**	*Constitutive*	1			
	*Locally Induced*	-0.27	1		
	*Systematically Induced*	-0.26	-0.05	1	
**Tolerance**		0.20	-0.11	-0.18	1

Tradeoffs are expressed in terms of negative correlations among the defences. Pearson correlation values are shown.

**Table 5:** Number of eggs (mean values ± SE) laid by T. solanivora on different potato varieties.

	Eggs
*Solanum phureja*	45.32 ± 5.37^A^
Criolla Colombia	37.50 ± 11.91^b^
Criolla Galeras	31.60 ± 8.42^b^
Criolla Guaneña	82.47 ± 14.84^a^
Criolla Latina	29.88 ± 7.40^b^
Criolla Paisa	41.73 ± 10.82^b^
*Solanum tuberosum*	20.85 ± 3.39^B^
Betina	27.44 ± 13.47^ab^
Capiro	17.60 ± 7.76^ab^
Esmeralda	12.56 ± 7.84^b^
Monserrate	22.92 ± 9.42^ab^
Nevada	11.75 ± 6.15^b^
Pastusa Suprema	27.67 ± 16.15^ab^
Puracé	19.89 ± 8.56^ab^
Roja Nariño	36.08 ± 18.74^a^
Rubí	11.45 ± 3.71^b^
Única	10.33 ± 5.27^b^
V1	5.25 ± 3.77^b^
Yungay	30.50 ± 11.34^a^

Different letters indicate differences following a Tukey–Kramer test: upper case letters denote differences between the two potato species, while lower case letters show differences within each species.

## Discussion

The premise that defences are costly and result from a tradeoff between allocating resources to defence and to growth and reproduction is one of the main assumptions of most plant defence theories [3], and is commonly invoked to explain both variation in the expression of constitutive defences [39] and the evolution of induced defences [40]. In this study, we did find variation in the levels of resistance and tolerance in response to tuber herbivory, a type of damage that is rarely studied. However, there seem to be no costs in terms of yield to the expression of either constitutive or inducible resistance. That is, we found no correlation between the expression of both types of resistance and yield in the absence of herbivores. The probability of detecting costs of plant defence may be low in agricultural systems given that most crops are typically provided with supplementary water and nutrients and are grown under insecticide-sprayed conditions without weeds, pests, or diseases. Actually, the logic underlying a defence-yield tradeoff is based on plants having limited quantities of resources available for both processes [2,41]. Therefore, under agricultural practices, fertilization and damage may weaken the strength of such tradeoffs.

In general, induced defences–like tolerance–have been assumed to be less costly, given that their expression is only activated after damage [40]. However, here, we did detect a cost in terms of yield from tolerance to tuber herbivory. This cost was detected in the form of a negative correlation between tolerance and yield in the absence of herbivory. In other words, potato varieties that have higher yields under controlled conditions have lower tolerance (*i*.*e*. are not able to compensate for the damage caused by tuber moths), while potatoes with lower yields can compensate and even have a higher production after damage than before damage (see Fig 1). To our knowledge no other study has estimated costs of tolerance to tuber damage. Under some scenarios, where herbivores are scarce or fluctuate, allocation and/or metabolic costs of maintaining mechanisms for regrowth could be sufficiently high to favour nontolerant plants [22,24]. Also, negative genetic correlations between tolerance and other fitness-affecting traits–like resistance–could slow or prevent the fixation of tolerance alleles within a population [22]. Potato growers in the Colombian Andes deal with a high pest pressure of belowground herbivores that are difficult to control. In potatoes, direct selection for palatability or nutrition has indeed reduced the amount of alkaloids in tubers [42,43]. Thus, selecting for tolerance traits could help guarantee good yields even under high pest pressure. Our data however suggest that plants with high yields in the absence of insects will be less tolerant and therefore, there will be a loss in yield due to higher pest pressure. We believe that the study of costs, benefits and tradeoffs of belowground defences represent an opportunity for future research.

The expectation of a tradeoff between resistance and tolerance is based on the logic that resources available for defence are limited and thus, high investment in resistance reduces those resources available for tolerance and vice versa [44]; however, this diversion should not be assumed. Alternatively, if herbivores prefer the most vigorous or nutritious plants–as proposed by the plant vigour hypothesis [45]–and, in turn, these plants happen to be more tolerant and less resistant then, this herbivore preference could also produce a negative correlation between resistance and tolerance [22]. In this study, we found no evidence of a tradeoff between resistance and tolerance to tuber herbivory. We also did not detect an oviposition preference for the more tolerant varieties. On the contrary we only found that females laid more eggs on the less resistant species. That is, there seems to be a positive relationship between oviposition preference and larval survival when comparing between the two potato species as expected by the “mother knows best principle” [46,47]. However, this relationship was not detected when comparing all varieties, suggesting that female preference at the variety level is driven by other mechanisms [48]. Some hypotheses have been proposed for why no tradeoffs between resistance and tolerance could exist [22]. A vigorous or well-provisioned plant could express both high tolerance and high resistance; this could be especially true in an agricultural context in which resources to plants are not limiting. Also, if defensive chemicals have other functions besides defence–for instance as storage proteins–then higher levels of protein storage could increase tolerance [22] and no tradeoff will be detected. The lack of this tradeoff in potatoes suggests that selective breeding in potatoes has succeeded in creating varieties able to express the whole range of allocation patterns in defence strategies, from varieties that express either high or low levels of both types of defences to varieties that express intermediate levels of both types of defences. Indeed, Leimu and Koricheva [12] found that in agricultural crops resistance and tolerance tended to be positively correlated while in natural systems there is a negative correlation between them.

It has been proposed that one mechanism that allows plants to tolerate herbivory is the reallocation of resources within the plant [22]. We tested this hypothesis and, surprisingly, found a positive correlation between aboveground compensatory growth and tolerance in terms of yield (Fig 2), that is, increased tuber weight after damage did not come at an expense of aboveground growth. This result suggests that, after damage, there are no shifts in the allocation of resources from aboveground to belowground tissues. In other words, our data suggest that plants that are able to compensate for belowground damage by creating bigger tubers were also inducing greater aboveground biomass. Observations of positive correlations between traits where tradeoffs, and hence negative correlations, are expected is difficult. However, a positive association between below- and aboveground biomass could be explained if tolerant varieties can increase nutrient acquisition, thus inducing a higher primary metabolism or photosynthetic rate [49]. Understanding the physiological mechanisms behind the tolerance response in potato remains a promising line of research because identifying and harnessing these traits will allow breeders to increase productivity under different pest pressures.

Some studies support the hypothesis that domestication has reduced the expression of defensive traits–particularly resistance–against multiple insects, and as a consequence herbivore performance on crops has increased [30,50]. One of the main challenges to crop protection is the evolution of counter-adaptations by herbivores and pathogens to crop resistance traits [51]. When resistance is ineffective against herbivores, either because of the domestication process or because of herbivore adaptation, the expression of tolerance may represent the only viable defence option for the plant to maintain yield in the presence of herbivory. Under this scenario, selecting for crop varieties that express high levels of tolerance or are even able to overcompensate in response to damage could provide a viable and inexpensive approach to increase crop productivity [21]. The success of sustainable crop production depends on our ability to select or create varieties that can allocate resources to both growth and defence. Thus, estimating the costs and tradeoffs among defences, and understanding their causes and consequences is still important in both basic and applied ecology. Ultimately, testing the limits of defence and growth under agricultural practices will provide an important feedback from applied science to basic plant-insect interactions theory and could provide a genetic basis for sustainable crop production [52].
